# “Why can’t they come up with a program that teaches people how to get high and go to work”: Structural vulnerability, personal substance use and recovery goals, and substance use treatment among sexual and gender minority persons (SGMP) in Los Angeles, California

**DOI:** 10.21203/rs.3.rs-10320093/v1

**Published:** 2026-10-08

**Authors:** Sid S. Ganesh, Rebecca P. Smeltzer, Gilbert A. Orta Portillo, Natalie Jefferson, Julia J. Koerber, Jacklynn Pham, Moranda L. Tate, Pamina M. Gorbach, Ricky N. Bluthenthal

**Affiliations:** University of Southern California; University of Southern California; University of Southern California; Cornell University; University of California, Los Angeles; University of Southern California; University of Southern California; University of California, Los Angeles; University of Southern California

## Abstract

**Introduction:**

Sexual and gender minority persons (SGMP) report disproportionately high rates of substance use in the United States, yet relatively low rates of treatment access and initiation. In this study, we examined substance use treatment experiences among SGMP who inject drugs in Los Angeles, California.

**Methods:**

We conducted 15 one-on-one qualitative semi-structured interviews with self-identified SGMP assigned male at birth. Eligibility included being assigned male at birth, reporting sex with men, and meeting at least one of the following: being a participant in either the NIH-funded mSTUDY or CANOP cohorts or reporting any opioid or injection drug use within the past two years. Data were collected at a community site associated with a syringe services program and LGBT+ service provider in Los Angeles, CA between May 2022 - February 2023. We employed inductive and deductive coding, using the intersectional risk environment framework and life course theory to develop our codebook and guide our thematic analysis.

**Results:**

Participants described motivations to decrease their substance use in order to use drugs more intentionally and reduce stigma. Disordered use was linked to financial and material insecurity, which participants perceived as inadequately addressed by treatment programs. Experiences of shame, punishment, conditionality, and loss of support were common in treatment settings. However, patients reported positive experiences with programs that addressed structural vulnerabilities (such as social isolation and financial hardship) and integrated multiple therapeutic modalities into care.

**Discussion:**

We found that participants view substance use recovery on a continuum that included reducing frequency and type of substance use and modifying routes of administration to reduce infection risk. These findings support integrating harm reduction-based therapeutic modalities into substance use treatment settings. Furthermore, our results emphasize how substance use treatment may be delivered to improve efficacy by 1) co-delivering structurally competent services, that address housing instability, social isolation, unemployment/underemployment, and professional development, 2) prioritizing patient directed, and centered care that incorporates harm reduction and shared decision making, and 3) providing integrated treatment that combines various treatment and therapeutic modalities to meet patients’ most pertinent needs.

## 1. BACKGROUND

### 1.1 Substance use among sexual and gender minority people (SGMP)

The 2024 National Survey on Drug Use and Health estimated that 73.6 million people aged 12 or older in the United States, or 25.5% of the population, used an illicit substance in the past year and further classified 48.4 million people (16.8%) as meeting the DSM-5 criteria for having a substance use disorder (SAMHSA, 2025). Among those with a substance use disorder, only one in five received treatment in the past year (SAMHSA, 2025). Sexual and gender minority people (SGMP), including lesbian-, gay-, bisexual (LGB) and transgender-identifying individuals, report disproportionately higher rates of substance use and substance use disorders compared to heterosexual and cisgender adults (SAMHSA, 2024; Goodwin et al., 2022; Schuler et al., 2018). National estimates from 2023 found that approximately half (48.8%) of LGB individuals aged 12 and older reported illicit substance use in the past year and 31.9% met the criteria for a substance use disorder (SAMHSA, 2024). Further, LGB adults were roughly six times more likely to use opioids and three times more likely to use stimulants such as cocaine (6.6%) and methamphetamine (2.5%) than heterosexuals (2.0% and 0.69%, respectively; Philbin et al., 2020). In a large sample of insured adults, the prevalences of opioid and cocaine use disorders were over three times higher among transgender versus cisgender patients (Hughto, Quinn, et al., 2021).

### 1.2 Inequitable substance-related and treatment outcomes among SGMP

Structural vulnerability refers to susceptibility to health-related harms that are driven by an individual’s location within greater social, economic, and political systems (Bourgois et al., 2017; Holmes, 2013; Quesada et al., 2011). Among people who use drugs (PWUD), structural vulnerability is reflected in unequal exposure to poverty, housing insecurity, material deprivation, criminal legal system involvement, and barriers to healthcare (Ganesh et al., 2025; Rhodes et al., 2012). SGMP who use drugs experience many of these intersectional risks at disproportionately high rates including housing instability (Zhang et al., 2025), economic marginalization (Schuler et al., 2021), incarceration (Harvey et al., 2022; Srivastava et al., 2024), mental health conditions (Bae et al., 2026; Schepis et al., 2026), and high risk practices for infectious disease (e.g., receptive syringe sharing, transactional sex; Guerra et al., 2020; Jenkins et al., 2025). Together, these intersectional and structural inequities contribute to increased risk for substance use-related morbidity and mortality among SGMP (Earnshaw, 2020).

Several evidence-based treatments to reduce opioid and stimulant use exist, including medication for opioid use disorder (e.g., buprenorphine, methadone; Rosenwohl-Mack et al., 2026; Carley & Oesterle, 2021; Kampman & Jarvis, 2015), bupropion and naltrexone for methamphetamine use disorder (Trivedi et al., 2021; Knight et al., 2019), and contingency management programs (Bolívar et al., 2021; Knight et al., 2019). These interventions are associated with decreased substance use and risk of negative substance use-related health outcomes including infectious disease (Hervera et al., 2023; Serota et al., 2023) and overdose (Wakeman et al., 2020; Larochelle et al., 2018). Despite their efficacy, access to, engagement, and retention in treatment is inequitable among PWUD. Structurally vulnerable PWUD face barriers to treatment including difficulty accessing healthcare services and delayed treatment (Ford et al., 2025; National Academies of Sciences, 2019), premature discharge from treatment (Chavez et al., 2025; Gaeta Gazzola et al., 2023), and inadequate treatment and support in healthcare settings (Adeniran et al., 2023). These may be intensified for structurally vulnerable SGMP who face intersectional barriers. For instance, SGMP report high unmet substance use treatment needs (Hughto, Restar, et al., 2021; Krasnova et al., 2021) and commonly experience stigma and discrimination due to sexual orientation and gender identify in treatment settings (Hillyard et al., 2026; Hughto, Restar, et al., 2021), which is associated with poorer treatment engagement and retention (Paschen-Wolff et al., 2024). These intersectionalities and structural vulnerabilities are apparent in the extant literature that documents disparities in treatment utilization among SGMP. For example, the 2023 National Survey on Drug Use and Health estimated that 0.4% of SGMP individuals with a substance use disorder sought substance use treatment in the past year and 6.3% did not seek treatment but thought they should get it (SAMHSA, 2024). Further, SGMP-affirming substance use treatment infrastructure is limited. In 2018, less than 1,700 of 14,000 substance use treatment facilities (federal, state, and local) in the U.S. offered methadone and/or buprenorphine treatment within programs tailored to SGMP clients (Paschen-Wolff et al., 2022).

We integrated structural vulnerability with the intersectional risk environment framework to examine how structural forces (immiseration, unemployment, and homophobia) interact among with PWUDs overlapping marginalized and minoritized identities (i.e., gender, sexuality, race, and socioeconomic class) to drive health risk (Collins et al., 2019). Specifically, for SGMP who use drugs, structural drivers such as drug criminalization, criminalization of homelessness, structural stigma and discrimination against queer and trans PWUD, and income inequality intersect with institutional and interpersonal social factors including homophobia, transphobia, violence, and trauma.

### 1.3 Objective

There has been limited research on structurally vulnerable SGMP’s experiences with substance use treatment and perceptions of recovery that directly assesses the role of the broader social environment in treatment experiences. In this qualitative analysis, we examined perspectives and experiences of substance use treatment identified by SGMP with lived experience of injection drug use. Results highlight how participants identified socioeconomic struggles as barriers to reducing substance use and offered insight into how recovery could be supported in SGMP communities.

## 2. METHODS

### 2.1 Study design

This research consisted of semi-structured qualitative interviews with 15 people to investigate patterns and motivations of injection drug use and polysubstance use, with a focus on opioids and stimulants, among SGMP living in Los Angeles, California. Our research team consisted of undergraduate students (NJ and JP), doctoral students (RPS, GOP, SSG, and JK), and faculty (PG and RNB), with RNB acting as the Principal Investigator of the study. Throughout the research process, we adhered to the consolidated criteria for reporting qualitative research (COREQ) checklist (Tong et al., 2007). The study was approved by the University of California, Los Angeles Institutional Review Board (study number 18–000876)

### 2.2 Participant Selection

Given diverse sexual and gender identities and the unique hardships faced per identity, we recruited those who identified as SGMP to highlight how one’s lived experience may influence their substance use and access to treatment. We utilized active and passive convenience sampling to recruit participants. Active recruitment occurred at a syringe service program in Los Angeles, California, and through direct referrals from the parent studies: mSTUDY, a NIDA-funded cohort of SGMP living with and without HIV (U01DA036267), and CANOP, a NIDA-funded cohort of opioid-using people who inject drug (R01DA046049) (Javanbakht et al., 2026; Ganesh et al., 2025). Passive recruitment included referrals from participants already enrolled in the study. Eligibility criteria included: aged 18 or older, assigned male at birth, report having sex with men, and any opioid or injection drug use within the past two years. Informed consent was obtained from all participants prior to participation, and each received $40 cash as compensation for their time.

### 2.3 Semi-Structured Interviews

The 45 to 90-minute interviews occurred in-person between May 2022 and February 2023. We used a semi-structured interview guide (see Appendix) based on existing qualitative and quantitative literature (Dangerfield et al., 2020; Boeri et al., 2011; Hser et al., 2007). The PI developed the interview guide using life course theory (Dangerfield et al., 2020; Elder et al., 2003), which posits that an individual’s life history, along with their social environment, plays a critical role in shaping their health and well-being (Elder et al., 2003). The interview guide explored family history of substance use, housing stability and living conditions, substance use behaviors, sexual and gender identity development, interpersonal relationships, health outcomes, and treatment experiences. Questions were asked at the levels of influence (i.e., individual, interpersonal, communal, and societal) and domain of influence (i.e., biological, behavioral, physical/built environment and healthcare system) as they relate to health outcomes and substance use.

Interviews were conducted by two team members– a doctoral student (GOP) and the PI (RNB), and aimed to uncover participants’ drug use motivations, patterns, and the broader social and environmental contexts shaping their drug use experiences. All interviews were audio-recorded using an iPad or iPhone. To protect confidentiality, participants were asked to select a pseudonym to be used throughout the interview. To ensure additional privacy, the research team later replaced these self-selected pseudonyms with new ones for data analysis and dissemination. All audio recordings were transcribed through Otter.ai and were manually verified and edited by the research team (RPS, GOP, JK, and AM). The files were uploaded to a HIPAA-compliant OneDrive for team analysis. Summaries of emerging ideas were generated after each transcript was reviewed.

### 2.4 Thematic analysis

We employed both inductive and deductive coding approaches, using interview domains to construct our initial codebook (Braun & Clarke, 2006). This codebook underwent multiple iterations, informed by discussions with team members responsible for transcript cleaning and by initial memos generated from four randomly selected transcripts (analyzed by RPS and SSG). Interview transcripts and the final codebook were uploaded to ATLAS.ti Mac Version 24.1.0. Transcripts were coded in dyads (RPS/GOP, RPS/JK, NJ/JP). Each coding pair met twice to assess coding consistency and address discrepancies, which were primarily minor variations in the length of coded text rather than substantive differences. Disagreements in coding were resolved through consensus-building discussions. This iterative process ensured that our analysis remained both data-driven and theoretically informed, enhancing the validity of our findings.

We (RPS, SSG, NJ) ran a co-occurrence analysis in ATLAS.ti on our code “treatment” and examined the most commonly co-occurring codes. They included adulthood and family dynamics, transitions and moving between locations, financial stability and instability, housing and material instability, stimulants, stopping use and reinitiation. We utilized the intersectional risk environment framework (Collins et al., 2019) to guide our thematic analysis. The intersectional risk environment framework examines differential drug- and health-related outcomes produced from intersecting systems of oppression and an individual’s life course (Collins et al., 2019). In this analysis, our findings are largely focused on structural vulnerability, as Collins et al. (2019) describes how structural vulnerability shapes the degree to which individuals are exposed to intersectional risks within a given environment. In this case, our findings describe how structural vulnerabilities shape exposure to intersectional risks among SGMP within the substance use treatment risk environment.

## 3. RESULTS

Participant characteristics (n = 15) are displayed in Table 1. Among participants, most identified as cisgender men (n = 13, 86.7%), gay (n = 9, 60.0%), non-Hispanic Black (n = 5, 33.3%), and were 39 years old on average (IQR 31–44). Over half were unstably housed in the past six months (n = 9, 60.0%) and had a diagnosed mental health condition (n = 9, 60.0%). Most used methamphetamine (n = 14, 93.3%), followed by cannabis (n = 9, 60.0%), and opiate use, primarily heroin (n = 5, 33.3%), and fentanyl (n = 5, 33.3%). Three quarters of the sample currently injected drugs (n = 11, 73.3%) and most had injected drugs at some point in their lifetime (n = 14, 93.3%). Participants shared their experiences with a variety of substance use treatment modalities including formal residential rehabilitative programs and informal, 12-step social support programs for people in recovery. From the interviews, we identified four major themes related to motivations for decreasing substance use; disordered substance use rooted in financial insecurity and material hardship; past treatment experiences that involved shame, punishment, conditionality and loss of social and material support; and treatment practices that facilitated reductions in substance use and meeting recovery goals by addressing structural vulnerabilities. We did not ask participants for their preferred pronouns and so, we refer to all participants by third person singular they/them/their pronouns in this manuscript.

### 3.1 Result 1: Motivations for decreasing substance use were more intentional/controlled use and reduced stigmata

Participants’ goals for substance use varied – some wanted to stop using drugs entirely, others wanted to change routes of administration, and/or reduce frequency of use. Participants delineated intentional use from disordered use. The former was seen as context-driven as opposed to dependence, or “need” comparing it to recreational alcohol use – importantly, on the spectrum of substance use, intentional use allowed participants to continue using substances to meet self-identified needs (social connection, anxiety or pain management) while not hindering their broader life goals (such as employment, family relationships). Alternatively, they described disordered substance use as undesirable because it made them too intoxicated to do what they wanted in their daily lives and eventually hindered their life self-determined goals.

For instance, Cyan wanted to use injectable drugs in a more intentional and controlled manner to mediate stigma, explaining:

*“I would like to say I wish [my drug use] would disappear because I feel like it held me back from a lot of things. I don’t know that it necessarily holds me back these days at the moment. I think I would like to be able to do it recreationally. When I mean, recreationally, totally in control. Totally not having to have it. It being something you enjoy, like wine or drinking at a party or something. It’s just something you do whenever the time comes up for it.”* -Cyan

Later Cyan reflected on how their substance use interfered with their productivity and interfered with their desire to become a software engineer.

On the other hand, Sienna had injected heroin for six months and stopped after witnessing their friend’s near-death overdose. Ever since, Sienna developed fear of the same outcome and began feeling uncomfortable with the physical effects of injection, including visible needle “track marks.” Sienna was able to stop injecting heroin to avoid the risks and currently uses other routes of administration and substances instead:

*“Because I had a friend that overdosed on heroin, and he almost died. But his wife was able to go ahead and call 911. And then they administered the Narcan and everything like that to him. So, I was scared that it might happen to me too, and I felt like the needle thing was getting a little bit too dirty for me. I felt like I had too many needle marks. So, I was like, ‘I should stop this.’ I prefer to just drink and smoke.”* - Sienna

In Sienna’s account, they saw their track marks as evidence of what could happen to them (i.e., overdose) if their current substance use trajectory continued uninterrupted. In this way, underneath reducing or shifting their patterns of use was the intention to shape their substance use trajectory and gain control over the potential risk of fatal overdose. It is worth noting the description of needles feeling “dirty” as an instance of internalized shame and responsibility associated with substance use related stigmata.

Participants expressed a desire to change use patterns in a way that better suited their goals to less frequently or completely stop injecting. For some, this meant a pattern of reduced substance use to meet more tangible, everyday goals, while for others this meant cessation of injection drug use to reduce negative health outcomes and reduce stigmata.

### 3.2 Result 2: Participants linked disordered substance use to financial insecurity and material hardship, which they felt was overlooked by treatment programs

Participants described concerns about structural issues with treatment programs that caused dissonance between personal substance use goals and the goals of the programs. Treatment programs focused on abstinence simultaneously ignored the structural drivers of functionality as described by participants (e.g., maintaining employment, consistent income, stable housing, daily routines), which led to hesitation in treatment initiation or re-initiation as it was viewed as impractical or unrealistic.

Jade appraised themselves negatively for no longer being able to maintain a job, have financial security, or afford rent after moving to Los Angeles (driven by structures and systems such as the stigmatization and criminalization of substance use as well as lack of affordable housing). While acknowledging their current lack of stability, they also verbalized a goal of wanting to take care of themselves and obtain permanent housing (compared to their current status of unsheltered homelessness):

*“I’ve always functioned. I’ve always had good jobs at the casinos, or the bars or whatever. I didn’t finish college but I’ve always had good money and I’ve always worked, did street shit on the side. I didn’t get dysfunctional or what I would consider dysfunctional, like now, until I moved to LA, when the pandemic hit. I haven’t worked. It’s been a three-year gap now in my employment…my life is so crazy right now. And just unorganized that I haven’t even looked for a job or even considered working again, I haven’t even thought about it… I wanna take care of myself…This is the longest I’ve been in the streets, you know?…And in [other state], I’d never have to worry about that. I could get an apartment for a week for like 140 bucks.”* - Jade

Jade’s goal to achieve financial and housing stability, and return to a “functional” state, did not align with existing treatment programs that prioritized abstinence. They envisioned a new type of “function program” that aligned with their goal of controlled substance use within daily routines, to support their ability to maintain employment and consistent income:

*“Why can’t they come up with a program that just teaches people how to get high and go to work, you know what I’m saying?… I wanna come up with one of those. Like a function program?”* - Jade

Similarly, Beige implied the underlying importance of basic needs and managing substance use in response to the question, *“So what do you think would help you continue moving forward, and help you grow and strengthen yourself?”* They viewed residential treatment programs as forcing abstinence in an environment that is not “regular living” or reflective of their current life situation, while not meeting the basic needs of people. Beige’s goal was to have financial and material security rather than stopping use and did not want to feel like a failure for that.

*“Basic things, money, a home, you know, stuff like that… just regular living… My sister keeps telling me that I should go to rehab, but I don’t really want to go to rehab, but she’s telling us that she’s a counselor and she says ‘we can get you into a good home’ and ‘I can get you into this and that.’ But I don’t really want to go there, do that. I did it when I was on parole, then it’s just like forced on me. I take it hard when it is forced on me… I don’t want to go in there and just fail and not make it, you know?”* - Beige

Alternatively, Umber viewed residential treatment settings as a place to eat, sleep, and rest for a month, but attending for a second time would not teach them new sustainable skills to decrease methamphetamine use that could be applied to their daily life living unsheltered on the street. Instead, they viewed attending residential treatment for a second time as taking away temporary basic needs from someone else.

*“I don’t know everything. But I know the basis that there’s someone who could use that bed probably more than me, I think. You know, if I’m just there just to have a place to sleep, to eat and just do nothing for 28 days. Excuse my language, but why the fuck am I there? There is someone else that’s dying, literally dying to get into a bed like that. To me, it’s no. So why? Why should I take an unnecessary bed when someone else actually needs it?”* – Umber

While participants showed a desire to change their use patterns to be more functional or controlled, they were ultimately discouraged by abstinence requirements and what they viewed as unrealistic treatment practices. The programs were not designed to address material, financial, and housing stability or prioritize personal agency in directing substance use goals. These frictions between program structures and individual substance use goals discouraged participants from attending a treatment program as it would not be effective in meeting their needs.

### 3.3 Result 3: Participants experienced shame, punishment, conditionality, and loss of social and material support during treatment

#### 3.3.1 While participants were motivated to attend residential treatment to avoid incarceration, they experienced punishment and conditionality by treatment programs

Multiple participants described instances when they were faced with the choice between incarceration or mandated treatment. While residential treatment programs provided initial support for decreases in substance use, punitive facility rules resulted in involuntary discharge, housing instability, and re-initiation of injection drug use.

After being arraigned, Ebony had to decide between court ordered substance use treatment at a residential rehabilitation facility or six years of incarceration. After making the decision to attend a residential treatment setting, Ebony spent just under five years at the same sober living facility, maintaining their abstinence from methamphetamine and heroin throughout that time. Upon accepting their identity as transgender and publicly coming out, rather than the facility providing or supporting gender affirming care, Ebony was required to leave and move to transgender inclusive transitional housing, which they could not afford to do.

“I came out of the closet [as transgender] for the first time ever - Knowing full well that I was going to lose everything. Knowing full well that I was going to end up with nothing in the end. And knowing full well that I’m not - probably not even transitioning in the end because it’s not within my privilege…Acceptance is the answer. That’s what I learned. Acceptance is the answer….I was doing recovery fully. I almost did - I almost had five years. I got asked to leave the sober living to transitional living, as I knew because it’s called [Transitional Living Program]….Well, I can’t afford it…Find me a place where you can live off of SSI.” – Ebony

Despite Ebony’s continued abstinence and following of facility rules, they were forced to leave because of their gender identity and unable to attend a transgender inclusive transitional living facility due to lack of financial security. Shortly after being discharged, Ebony returned to the neighborhood where they previously purchased drugs, which led to re-initiation of injection methamphetamine and heroin use and also acted as the place where they were able to obtain hormones to support in their medical transition, as they were unable to receive gender affirming care elsewhere:

“It was a matter of fucking going down to [redacted] Park where I fuckin’ went to get my hormones. Same spot. Get hormones or you get [methamphetamine and heroin].” – Ebony

While not faced with court ordered treatment, another participant’s motivation for treatment and decreased opioid and methamphetamine use was to prevent themselves from having to live unsheltered on the street and to avoid future incarceration. To achieve this, Pewter entered detox many times but would often return to use. At one time, the director of the detox who had an existing relationship with Pewter gave an ultimatum of attending residential rehabilitation, which Pewter agreed to and felt that they were making progress through the support of the program and peer support. After four months of residing at the program and maintaining abstinence, Pewter was caught fraternizing with another resident, which resulted in them being discharged from the facility, and not being allowed to graduate or transition to the program’s sober living housing. Because of this, Pewter returned to unstable housing conditions and the eventual re-initiation of injection substance use.

“I never made it to Skid Row in a tent. Because luckily, I had, you know, people that I can crash pad and stuff like that, you know, and I was always doing like little crimes here and there, going to jail and prison and stuff like that. But at that time, you know, I started to realize at the end of that, that this is not what I want. You know what I mean? I was like, ‘I gotta do something, I gotta do something.’ And I didn’t want to live on the street…So I attempted to put myself into treatment. Now it took a few times, you know, I’d go to detox, and then I’d leave…when I was 32, I went into a treatment center and…like I said, I had been there before, to the detox many, many times. I’d say about 15 times. And the guy, the director for the detox said, ‘Pewter, look man, I’m gonna admit you, okay? I’ll take you in, but you’re going to stay for the fucking residential. You know or else I’m not gonna let you into detox.’ He’s like, ‘You’re gonna fucking stay bro, because you know, this, and this, and that.’ So I did, and I felt I was fortunate because I was in there with a group of people that were - we just - everybody just clicked. Everybody got along. Everybody clicked. I ended up having like a little romance, you know, in there. But it was like a good group to get sober to like, take that next step, that change, you know? Unfortunately, I did not complete - though technically, I did stay there 4 months…Yeah so I was about four months in. I, you know, I had a slip up. I got kicked out for fraternizing with the girl. And then when I got kicked out, you know, when you get kicked out, you don’t get the opportunity of like that, you know, if you were to graduate, they would set you up with one of their sober livings.” – Pewter

In this way, participants were motivated to attend residential treatment to avoid incarceration; however, in treatment they experienced punishment and conditionality that served as impediments to their personal substance use and recovery goals.

#### 3.3.2 Negative experiences with abstinence-based, 12-step group experiences were associated with shame, emotional distress, and discomfort with public disclosure.

Participants reported prior negative experiences with 12-step program settings. These included feelings of guilt, embarrassment, and shame in 12-step programs as barriers to treatment re-initiation. The methods used within the 12-step programs, such as publicly disclosing traumatic or private information in a group setting, embarrassed participants to a degree that discouraged them from continuing treatment at all.

Mauve’s experience with 12-step programming was overwhelming and emotionally distressing, as it required recounting traumatic experiences publicly without supportive individuals or tools to help manage those emotions. Revisiting painful memories made Mauve feel worse during the process, leading them to view the program as a negative experience.

*“…I work[ed] 12 steps, and all it did was make me feel worse […] In 12 steps, we had to do that soberly. I wanted to kill myself, like it was bad. I say that laughing, but like really honestly… Like cold turkey… - sober…, oh my gosh, everything that you ever compartmentalized, and you threw away and thought you got rid of? No, it’s running faster than you, and if you decide to stop running[…] it catches up with you… There’s nothing there to love you, or distract you, you’re honest and you’re around, exposing yourself to everyone in this room, and they’re all looking at you cry. It’s crazy. That makes me feel horrible. I felt like someone that was…starting to think there’s wrong with me in front of a whole auditorium full of people, you know? But it was me… doing it to myself, telling on myself, like it’s just crazy. I don’t like that feeling, I don’t like that cult. I feel like it’s a cult. […] you know what honestly- not one of my sponsors that I had ever lived on, you know what I mean? All of them like cut corners, or like they’ll relapse. Nope, I’ve never been a person who’s serious about the 12 steps”* - Mauve

Similarly, Cyan described feeling overwhelmed with guilt and embarrassment during an Alcoholics Anonymous (AA) meeting after crying while recounting their substance use journey in front of the group. This experience created a strong emotional association with shame and discomfort in group settings, discouraging them from returning even after being offered support through sponsorship.

“AA meetings are a little different because after I used a few times, I went to look for a meeting here in [neighborhood] and all I remember was I cried the entire time throughout the meeting. I couldn’t stop. I just felt really guilty. And it was, it was really hard to - just I don’t like crying in front of people. And I just couldn’t stop. And, but I didn’t - they gave me phone numbers for these people who want to be sponsors, but I really didn’t want to get back to all -I just didn’t want to do that all. I just felt really bad and embarrassed. So I never went again, like that.” –Cyan

While Cyan avoided further participation in 12-step programs due to the emotional vulnerability required, they did not reject all forms of formal treatment. They ultimately sought help from a psychiatrist, suggesting a preference for individualized care over peer-based recovery models.

The emotional disclosures required in 12-step programs left some participants feeling exposed, ashamed, and unsupported, which discouraged them from continuing or returning to those settings. While these experiences shaped aversion to peer-based, abstinence-only models, they did not always lead to a complete rejection of treatment. Instead, participants emphasized the need for emotional readiness and the ability to choose treatment environments aligned with their goals in mind. These factors played a central role in shaping how participants thought of reinitiating care in the future.

### 3.4 Result 4: Treatment programs that focused on addressing financial and material insecurity and other structural vulnerabilities (i.e., social isolation) facilitated reductions in substance use and meeting recovery goals

Participants found that the integration of social support increased their motivation for treatment reinitiation and retention. Both family support and “found family” support from peers in these programs helped participants initiate treatment as well as stay consistent and focused on their treatment goals.

#### 3.4.1 The role of social support in recovery

In Beige’s experience, they describe how intergenerational family dynamics influenced their experience with rehabilitation programs.

*“They sent me to rehab out here. My mother… was still into drugs and drug dealing… so my grandmother, I lived with my grandmother, and she sent me to rehab. Grandma, Grandpa, they drove me out here and they dropped me off here at the rehab and… they would come visit me and bring me cigarettes, you know, when they could. They really - they didnť want me to go into that, into that life.”* - Beige

Through consistent visits and small gestures, like bringing cigarettes, Beige’s grandparents demonstrated their ongoing support for Beige, which Beige identified as a critical factor in shaping their trajectory with substance use.

Sienna identified the role of familial support in treatment selection, the impact of crowd sizes and perceived quality of care, and the importance of communicating one’s comfort and willingness to engage in treatment as key components of what drives someone’s experience with substance use treatment. In vocalizing their concerns, Sienna asserts their agency by expressing their dissatisfaction with one treatment facility and later emphasizing their willingness to engage in a different facility, highlighting how the fit of the treatment environment can positively affect one’s experience and their treatment process.

*“My parents were looking into like a place for me to go to. So first, they put me in [recovery center in Los Angeles]. And then after a while, I didnť like it. I said, ‘I really donť like [recovery center in Los Angeles]. There’s a lot of people here,’ and I just didnť like the crowd of people. It was just like too many people, and they were doing it more in bulk than in quality. Yeah. So I was like, ‘Okay, fine. I’ll be willing to go to a different place, if you guys can find a different place.’ So my parents researched and then they found the [other location]. It was good.”* – Sienna

Sienna and Beige’s familial support systems facilitated their entry into formal rehabilitation programs. Beige’s grandparents removed barriers to treatment such as transportation and isolation by driving them to the program and providing companionship when possible, while Sienna’ family prioritized Sienna’s comfort in the treatment program to ensure that they continued treatment in a suitable setting.

Unlike Sienna and Beige whose families were involved in treatment, Topaz felt the isolation and loneliness of navigating their treatment trajectory without social support.

*“Get the proper treatment. The support group, get out of drugs. Start taking care of myself. […]Something encouraging, like a family. […] I lost all the family I had. The family I had left. […]. It would be helpful to find a support group. […] You can have friends that are like a brother to you. It helps. Oh really it does. Iťs the closeness.”* – Topaz

Topaz identified this lack of support as a vulnerability in their treatment, noting that connection helps with taking care of themselves, and external encouragement from trusted social actors uniquely shapes substance use trajectories. Further, Topaz points to how close social relationships can be fostered and organized in recovery settings through groups, and active relationship building so that participants can provide mutual support towards a shared recovery goal.

Lack of social support has long since been identified as a structural vulnerability. Conversely, here participants identified the ways in which having social support via family and/or friends expanded their capacity to engage with treatment. In this way, participants’ relationship with, and attitude towards, treatment was facilitated by mutually reinforcing internal and external support systems.

#### 3.4.2 Integrating multiple therapeutic modalities within recovery and treatment services

Several participants reported having positive experiences with treatment programs that offered integrated and whole-person types of support, including mental health prioritization, therapeutic communities, and contingency management-style interventions.

After voicing their need for a supportive rehabilitation facility and later transitioning to one, Sienna described how the emphasis on community was integrated into the care they received.

*“The treatment model was a therapeutic community, and then you would see the therapists at least one time a week, and then there would be certain drugs that they wouldnť allow you to have, obviously not allow you to have meth, heroin, and all the legal marijuana and all those other drugs, but they would allow you to have like, Zoloft [Sertraline, a Selective Serotonin Reuptake Inhibitor (SSRI)], and then also, what was the other one? And Latuda [lurasidone, an anti-psychotic].”* - Sienna

With their recounting, Sienna underscored the importance of having a structured treatment environment (i.e., no illicit substances or cannabis, weekly therapy at minimum) that integrated multiple evidence-based modalities, in this case, therapy and medication management treatment.

Ash compared treatment experiences with other programs in past, and described how and why they found that contingency management-style treatment models were suited to their recovery trajectory:

*“I went to one program voluntarily because I’ve been to jail for selling drugs four times…So their whole thing was you go to counseling, iťs not a 12-step program. I’ve met enough 12-step people that you know they’re preachy about how bad you are, but then they’re gonna see you as a joke. But… it wasnť 12-step and then [they] pay you in gift cards every [time] you tested clean. Test four times a week. … I get to - collecting these gift cards throughout your time period within. It was a 15-month period or have them hold the [gift cards until] the end. I had let them hold it to the end. I had $1,600 worth of gift cards at the end cuz I never touched them during it was kind of an incentive to be clean and it just felt good.”* – Ash

Additionally, contingency management incentives motivated Ash to stay sober by receiving financial incentives and appreciating the continued supportive relationships from staff members and fellow patients. Ash continued:

*“Not only for the gift cards, but there was the time that I spent in the group meetings and stuff. I tried to take advantage of that. The mediator and the two guys… that worked at the program. They were really nice. I took their contact info, not the mediator, but the two. I text them every now and then and see how they are.”* -Ash

These positive experiences highlighted the importance of being able to access a treatment program that therapeutically aligned patienťs recovery goals and needs, which varied in different therapeutic models.

## 4. DISCUSSION

The core contribution of this study is to expand the existing corpus of research describing substance use, treatment, and recovery as a continuum by illustrating the covert ways in which abstinence continues to be embedded into current treatment delivery and practices of care; and to provide evidence-based implications for practice and health service delivery. We found that: participants viewed and experienced substance use along a continuum, ranging from disordered to intentional and were motivated to alter their substance use so it was more intentional. However, they identified how patterns of disordered use were closely tied to structural vulnerabilities such as financial precarity/insecurity, material hardship, and social isolation. Psychosocially, multiple participants described how prior treatment experiences were characterized by shame, punishment, and conditionality, while treatment programs that addressed structural vulnerabilities (such as strengthening social support) were more effective in facilitating reductions in use and meeting personal substance use and recovery goals.

Our findings have the following implications to improve substance use treatment delivery for SGMP: 1) co-delivering structurally competent services: prioritize co-delivery of services that address participants’ structural vulnerabilities specifically, housing instability, unemployment/underemployment, professional development, and social isolation, 2) patient directed, and centered care: prioritize harm reduction and shared decision making, and 3) integrated treatment: integrate various treatment and therapeutic modalities to meet patients’ most pertinent needs.

Our findings suggest that co-delivering services that prioritize addressing participant structural vulnerabilities specifically, material needs insecurity, housing instability, unemployment/underemployment, professional development, and social isolation, alongside substance use treatment may support patients’ recovery. Participants described how their disordered substance use emerged from layered forms of vulnerability embedded within an intersectional risk environment, rather than as an isolated individual behavior. Financial and material insecurity–including unstable housing, strict abstinence-focused programs, and neglect of basic needs, like food– intersected with social marginalization and limited access to supportive services to produce conditions in which substance use may be out of the control of the clients. Participants emphasized that these structural vulnerabilities were mutually reinforcing, constraining agency and shaping risk in ways that behavior-focused treatment cannot address alone. Treatment programs were perceived as overlooking this broader risk environment by prioritizing abstinence and individual responsibility while failing to acknowledge and address the material and social conditions that sustained substance use. As a result, participants viewed intervention efforts as misaligned with their lived realities, reproducing vulnerability rather than addressing and preventing it. Providing structurally competent care via basic needs, such as housing, food security, employment and healthcare, into treatment facilities for people who use drugs may facilitate treatment engagement and retention while directly shaping substance use disorder outcomes. The concept of “radical hospitality” provides a framework for operationalizing basic needs integration through unconditional acceptance, low barrier access and generous provision of material supports (Wenger et al., 2024).

Housing instability undermines retention and increases re-initiation of substance use, alternatively Housing First models result in earlier housing entry, lower program exit, and decreased substance use compared to abstinence-focused treatment first models (Davidson et al., 2014; Padgett et al., 2011; Tsemberis et al., 2004). For example, in New York, individuals experiencing chronic homelessness placed in Housing First programs with high fidelity to non-judgmental client-centered care had significantly lower odds of stimulants or opioids use after one year of housing placement (OR: 0.17, 95% CI: 0.07, 0.57; Davidson et al., 2014). Beyond housing, unmet vocational and employment barriers exert among the largest measurable effects on subsequent drug-use improvement. Prospective cohort research shows that a greater percentage of matched needs (e.g., medical, mental health, family, vocational, and housing) predicts reduced substance use (Friedmann et al., 2004). Integrated service models have addressed basic needs and have demonstrated measurable, clinically significant outcomes across multiple domains. For example, the Comprehensive, Continuous, Integrated System of Care model, which integrated supportive housing, vocational training, and linkage to treatment, resulted in significant improvements in housing stability, employment rates, mental health symptoms, and reduced substance use patterns at 6- and 12-month follow-ups among individuals with co-occurring mental health and substance use disorders (Moore et al., 2009; Harrison et al., 2008).

While prior research demonstrates that addressing housing and employment improved substance use outcomes, participants in our study also emphasized that maintaining social relationships and addressing social isolation through treatment were a priority. Participants described effective treatment as programs that: 1) integrated patient advocacy to center their autonomy during treatment (whether family, friends, or community based); and 2) treatment modalities that prioritized social support. SGMP individuals who use substances shared that their nuanced relationships, such as the support of peers within treatment settings as well as family members who encouraged treatment via continuous presence in their life and actively facilitated access to treatment that best suited their preferences. These findings suggest that the benefits of integrated service models must also foster interpersonal support, in addition to addressing housing and material needs, to improve personal recovery outcomes among SGMP who use drugs. This is consistent with evidence from multiple recent systematic reviews that social recovery interventions, such as peer recovery support and recovery coaching, constitute a form of recovery capital that supports sustained and improved substance use treatment outcomes (Eddie et al., 2025; Bunaciu et al., 2024; Sinclair et al., 2024).

Our findings underscore how treatment protocols that restrict patient autonomy lead to negative perceptions of the effectiveness of treatment and future reengagement. In practice, evidence-based approaches that center and prioritize autonomy in treatment, as aligned with the principles of harm reduction, are evidence-based for effective implementation among SGMP populations (Hawk et al., 2017). Participants emphasized that harm reduction approaches to substance use (i.e., using less or using differently) and treatment (i.e., non-judgmental non-stigmatizing, and trauma-informed care) were important components of effective care. Participants’ reflections on prior experiences in residential treatment settings and 12-step recovery support programs highlighted that is was common to encounter abstinence-only frameworks in treatment and support settings. Participants saw these approaches as a barrier to future treatment engagement and emphasized that their personal goals focused on reducing risk through controlled or intentional use, rather than pursuing abstinence. Participants shared that forceful recovery practices were unsustainable as they only temporarily stopped using substances when forced to via strict treatment facility policies. When cessation came at the expense of daily function (work responsibilities, relationships, and personal agency), frictions occurred and engagement in treatment decreased (Ford et al., 2025). Further, participants described prominent feelings of guilt, embarrassment, and shame due to mandated group disclosure throughout 12-step programming. The heavy emotional vulnerability of sharing one’s experiences with substance use in 12-step group settings has been previously critiqued by both clinicians (Mendola & Gibson, 2016), and by people who use drugs related to its focus on admitting powerlessness, inability to control substance use, and lack of strength-based perspectives (Vederhus et al., 2020). While not explicitly named in our study, in other qualitative studies, individuals with the intersectional identity of being a gay or bisexual Black men who participated in peer recovery groups, have shared that group identity dynamics (i.e., if the group is predominantly white or straight) can make a space less welcoming for sharing, and discourage participation (Hussen et al., 2021; Jerome & Halkitis, 2014). Mutual help programs, such as peer group recovery model support, that incorporate cognitive-behavioral and motivational interviewing principles, such as SMART recovery, tailored to SGMP may more effectively support changes in substance use while simultaneously acknowledging intersecting structural vulnerabilities that impact mental health (McGeough et al., 2023).

Participants found that integrated treatments which combine multiple therapeutic modalities (medication, psychosocial, harm-reduction) are more effective in meeting patients’ needs than single delivery mechanisms. Treatment settings that integrated multiple therapeutic modalities such as psychotherapy (individual and group-based), contingency management (CM), medication management, along with offering residential and outpatient options allowed participants to find treatment pathways that aligned with their personal substance use and recovery related goals. Co-delivery and integration are evidence-based mechanisms for substance use treatment (Appa et al., 2026; Beshirs et al., 2026; Chatterjee et al., 2025; Appa et al., 2024; Komaromy et al., 2023; Bolívar et al., 2021; Knight et al., 2019; Reback & Shoptaw, 2014). In a systematic review of interventions to address substance use and sexual risk practices among men who have sex with men (MSM), Knight et al., (2019) found that while pharmacological interventions had limited efficacy in reducing methamphetamine use, the integration of psychosocial interventions such as weekly substance use counseling sessions were more efficacious at both methamphetamine use reductions and decreases in the number of sexual partners and instances of unprotected receptive anal sex (Colfax et al., 2011; Santos et al., 2016). Likewise, CM programs that combined individual MSM-tailored cognitive behavioral therapy and programs that integrated MSM-specific social support groups both had greater reductions in both methamphetamine use and high-risk sexual practices compared to traditional CM programs alone (Reback & Shoptaw, 2014; Shoptaw et al., 2008, 2005). The integration of CM programs and psychotherapy has also been proven to be effective in maintaining adherence to other pharmacotherapies common among PWUD including medication for opioid use disorder (Bolívar et al., 2021), HIV prevention and treatment medications (Appa et al., 2026; Steiner et al., 2025), and antibiotics for serious injection-related infections (Appa et al., 2024). The integration of multiple treatment modalities into existing harm reduction-based interventions such as harm reduction housing initiatives (Chatterjee et al., 2025; Komaromy et al., 2023) and street medicine programs (Beshirs et al., 2026) also increase engagement with substance use treatment and medication for opioid use disorder adherence.

### 4.1 Limitations

This study is not without limitations. First, interviews were recorded outside, and some audio-technical difficulties resulted in not being able to transcribe small parts of sentences. Each recording was reviewed by multiple people to recover as much text as possible, and any gaps were carefully reflected in the transcripts. Next, study findings may not be generalizable to other SGMP and/or PWID outside of Los Angeles as we used convenience sampling to recruit participants. The goal of this study was to examine injection trajectories and in doing so, treatment intentions and experiences emerged as a significant and participant-directed finding via our non-directive and semi-structured interviewing approach. Hence, inclusion criteria were not limited to participants who had experiences with specific treatment settings, and we did not probe extensively for further information about treatment. Additionally, we reported on the themes that most emerged from this dataset as determined by co-occurrence analyses. While sexual identity and treatment experiences did not significantly co-occur in our analyses, factors associated with intersectional risk (Collins et al., 2019), including housing, material, and financial stability did frequently co-occur. We expand existing literature on substance use treatment by highlighting the importance of intersecting vulnerabilities common among SGMP in relation to motivations for, and facilitators to, treatment. Finally, while this study focused on motivations for decreasing substance use and treatment experiences, we did not follow these participants on their journey to accessing treatment, nor did we assist in linkage to substance use treatment.

### 4.2 Conclusion

Our findings contextualize motivations for decreasing substance use as well as perspectives and experiences of substance use treatment among a population demonstrating experiences with significant intersectional stressors, including financial, material, and housing instability; risk for incarceration; mental health symptoms; and SGMP identity. These findings highlight the importance of aligning substance use treatment with individuals lived experiences, personal goals, and material and basic needs. Participants emphasized the need for nonjudgmental, integrated care models that mindfully address structural factors, reduce stigma, and promote social support and retention in treatment. Findings underscore the need for patient-directed and non-punitive harm reduction-based approaches to substance use treatment among SGMP. This entails providing care that does not mandate abstinence, avoids shaming or isolating participants, and refrains from exposing them to emotionally distressing practices. Instead, treatment programs and settings should adopt low-threshold access while prioritizing trauma-informed communication, patient-led treatment decisions, and respect for diverse individuals’ readiness and goals.

## Figures and Tables

**Table 1:** Participant characteristics and substance use behaviors among sexual and gender minority persons in Los Angeles, CA

Variable	Mean (IQR) or Frequency (%)

*Participant Characteristics*	
Age	38.7 (31, 44)

Race/Ethnicity	5 (33.3%)
Non-Hispanic Black	4 (26.7%)
Hispanic/Latine	1 (6.7%)
American Indian Alaska Native	1 (6.7%)
Non-Hispanic White	4 (26.7%)
Did not report race/ethnicity	

Gender Identity	13 (86.7%)
Male	1 (6.7%)
Gender Non-Conforming	1 (6.7%)
Other Gender Identity	

Sexual Identity	9 (60.0%)
Gay/Homosexual	4 (26.7%)
Bisexual	1 (6.7%)
Heterosexual	1 (6.7%)
Other Sexual Identity	

Houseless/Unstably Housed in the Last 6 Months	9 (60.0%)

One or More Diagnosed Mental Health Conditions	9 (60.0%)

*Substance Use Behaviors*	
Current Substance(s) Used	14 (93.3%)
Methamphetamine	4 (26.7%)
Powder Cocaine	3 (20.0%)
Crack Cocaine	5 (33.3%)
Heroin	5 (33.3%)
Fentanyl	4 (26.7%)
Opioid Pills	4 (26.7%)
Goofball (heroin & methamphetamine combined)	2 (13.3%)
Speedball (heroin & cocaine combined)	9 (60.0%)
Cannabis	2 (13.3%)
Gamma-hydroxybutyrate	

Injection Use	14 (93.3%)
Ever Inject	11 (73.3%)
Currently Inject	

## References

[1] Adeniran E, Quinn M, Wallace R, Walden RR, Labisi T, Olaniyan A, Brooks B, Pack R. A scoping review of barriers and facilitators to the integration of substance use treatment services into US mainstream health care. Drug Alcohol Depend Rep. 2023;7:100152. 10.1016/j.dadr.2023.100152. PMC10105485 37069961

[2] Appa A, Baral S, Stein B, Knight K, Gandhi M, Coffin P, Martin M. The IMPACT (Infection Management Plus Addiction Care Together) Pilot: A Case Series of Combined Contingency Management for Substance Use Disorders and Antibiotic Adherence in the Hospital Setting. J Addict Med. 2024;18(2):138–43. 10.1097/ADM.0000000000001254. PMC10939948 38109334

[3] Appa A, Steiner G, Baral S, Riley E, Suchman L, Roberts K, Knight K, Murphy M, Chamie G, Shoptaw S, Coffin PO, Gandhi M. Integrating HIV and Stimulant-Use Disorder Treatment: A Pilot Implementation Effectiveness Trial of Contingency Management in HIV Care. Clin Infect Diseases: Official Publication Infect Dis Soc Am. 2026;82(4):e728–34. 10.1093/cid/ciag006. PMC13131947 41505433

[4] Bae D, Leventhal AM, Harlow AF, Han D-H, Krueger EA, Barrington-Trimis JL, Conn BM, Cho J. Associations of Sexual Identity and Gender Identity with Substance Use and Mental Health in Young Adulthood. Subst Use Misuse. 2026;61(4):609–19. 10.1080/10826084.2025.2569792. 41102932

[5] BeshirsP, GaneshS, BluthenthalR, ConnerB, HerreraA. (2026). Healthcare First: Delivering Immediate, Non-Contingent, and Coordinated Services Through the Street Medicine Model (Rs. 3.rs-8233628). Research Square. 10.21203/rs.3.rs-8233628/v1

[6] Boeri M, Whalen T, Tyndall B, Ballard E. Drug use trajectory patterns among older drug users. Subst Abuse Rehabilitation. 2011;2:89–102. 10.2147/SAR.S14871. PMC3130350 21743792

[7] Bolívar HA, Klemperer EM, Coleman SRM, DeSarno M, Skelly JM, Higgins ST. Contingency Management for Patients Receiving Medication for Opioid Use Disorder: A Systematic Review and Meta-analysis. JAMA Psychiatry. 2021;78(10):1092–102. 10.1001/jamapsychiatry.2021.1969. PMC8340014 34347030

[8] Bourgois P, Holmes SM, Sue K, Quesada J. Structural Vulnerability: Operationalizing the Concept to Address Health Disparities in Clinical Care. Acad Medicine: J Association Am Med Colleges. 2017;92(3):299–307. 10.1097/ACM.0000000000001294. PMC5233668 27415443

[9] Bunaciu A, Bliuc A-M, Best D, Hennessy EA, Belanger M, Benwell C. Measuring recovery capital for people recovering from alcohol and drug addiction: A systematic review. Addict Res Theory. 2024;32(3):225–36. 10.1080/16066359.2023.2245323. PMC11262562 39045096

[10] Carley JA, Oesterle T. Therapeutic Approaches to Opioid Use Disorder: What is the Current Standard of Care? Int J Gen Med. 2021;14:2305–11. 10.2147/IJGM.S295461. PMC8184146 34113160

[11] Chatterjee A, Rapisarda SS, Silcox J, Zaragoza S, Summers C, Rolles A, Kosakowski S, Green TC. Now that I’m here, I actually have the stability and the time to really think about stuff like that: Perspectives on medications for opioid use disorder in low-threshold harm reduction housing. J Subst Use Addict Treat. 2025;179:209810. 10.1016/j.josat.2025.209810. PMC12668795 41015388

[12] Chavez CLJ, Peltier MR, McKee SA. Sex differences in the impact of social determinants of health on substance use disorder treatment outcomes. Biology Sex Differences. 2025;16(1):56. 10.1186/s13293-025-00734-3. PMC12281950 40696464

[13] Colfax GN, Santos G-M, Das M, Santos DM, Matheson T, Gasper J, Shoptaw S, Vittinghoff E. Mirtazapine to Reduce Methamphetamine Use: A Randomized Controlled Trial. Arch Gen Psychiatry. 2011;68(11):1168–75. 10.1001/archgenpsychiatry.2011.124. PMC3437988 22065532

[14] Collins AB, Boyd J, Cooper HLF, McNeil R. The intersectional risk environment of people who use drugs. Soc Sci Med. 2019;234:112384. 10.1016/j.socscimed.2019.112384. PMC6719791 31254965

[15] Dangerfield DT, Heidari O, Cooper J, Allen S, Lucas GM. Motivations for Opioid and Stimulant Use among Drug Using Black Sexual Minority Men: A Life Course Perspective. Drug Alcohol Depend. 2020;215:108224. 10.1016/j.drugalcdep.2020.108224. PMC7502465 32777690

[16] Davidson C, Neighbors C, Hall G, Hogue A, Cho R, Kutner B, Morgenstern J. Association of Housing First Implementation and Key Outcomes Among Homeless Persons With Problematic Substance Use. Psychiatric Serv. 2014;65(11):1318–24. 10.1176/appi.ps.201300195. 25022344

[17] Earnshaw VA. Stigma and substance use disorders: A clinical, research, and advocacy agenda. Am Psychol. 2020;75(9):1300–11. 10.1037/amp0000744. PMC8168446 33382299

[18] Eddie D, O’Connor JB, George SS, Klein MR, Lam TCS, Abry A, Hoffman LA, Hennessy EA, Vilsaint CL, Kelly JF. Peer Recovery Support Services and Recovery Coaching for Substance Use Disorder: A Systematic Review. Curr Addict Rep. 2025;12:40. 10.1007/s40429-025-00645-8. PMC12811009 41551498

[19] Elder GH, Johnson MK, Crosnoe R. (2003). The Emergence and Development of Life Course Theory. In Mortimer J. T. & Shanahan M. J., editors, Handbook of the Life Course (pp. 3–19). Springer US. 10.1007/978-0-306-48247-2_1

[20] Ford A, Ganesh SS, Ninh K, Gould EE, Page CJ, Gonzalez D, Ceasar K, Corsi RC, K., Bluthenthal RN. It’s Not Just One Decision, It’s a Whole Lifestyle Change: Mixed Methods Analysis of Factors Associated with Readiness to Change Substance Use Patterns Among Community Recruited People Who Inject Drugs. Subst Use Misuse. 2025;60(12):1787–98. 10.1080/10826084.2025.2519400. PMC12233208 40569245

[21] Friedmann PD, Hendrickson JC, Gerstein DR, Zhang Z. The effect of matching comprehensive services to patients’ needs on drug use improvement in addiction treatment. Addiction (Abingdon England). 2004;99(8):962–72. 10.1111/j.1360-0443.2004.00772.x. PMC2761625 15265093

[22] Gaeta Gazzola M, Carmichael ID, Christian NJ, Zheng X, Madden LM, Barry DT. A National Study of Homelessness, Social Determinants of Health, and Treatment Engagement Among Outpatient Medication for Opioid Use Disorder-Seeking Individuals in the United States. Substance abuse. 2023;44(1–2):62–72. 10.1177/08897077231167291. 37226909

[23] Ganesh SS, Gould EE, Smeltzer RP, Goldshear JL, Huh J, Ceasar RC, Bluthenthal RN. You don’t have the right resources to let it hurt: How structural vulnerabilities shape opioid withdrawal experiences among a community sample of people who inject drugs in Los Angeles, California. Harm Reduct J. 2025;22(1):82. 10.1186/s12954-025-01223-x. PMC12087061 40389990

[24] Goodwin SR, Moskal D, Marks RM, Clark AE, Squeglia LM, Roche DJO. A Scoping Review of Gender, Sex and Sexuality Differences in Polysubstance Use in Adolescents and Adults. Alcohol Alcohol. 2022;57(3):292–321. 10.1093/alcalc/agac006. PMC9630122 35284931

[25] Guerra FM, Salway TJ, Beckett R, Friedman L, Buchan SA. Review of sexualized drug use associated with sexually transmitted and blood-borne infections in gay, bisexual and other men who have sex with men. Drug Alcohol Depend. 2020;216:108237. 10.1016/j.drugalcdep.2020.108237. 33091811

[26] Harrison ML, Moore KA, Young MS, Flink D, Ochshorn E. Implementing the Comprehensive, Continuous, Integrated System of Care Model for Individuals with Co-Occurring Disorders: Preliminary Findings from a Residential Facility Serving Homeless Individuals. J Dual Diagnosis. 2008;4(3):238–59. 10.1080/15504260802072305.

[27] Harvey TD, Hughto JMW, Clark KA. Disparities in Incarceration and Its Health Implications in the United States, 2010–2020. LGBT Health. 2022;9(2):81–93. 10.1089/lgbt.2021.0090. Availability of Accessible Representative Health Data to Examine Sexual and Gender Minority. PMC9022180 35167353

[28] Hawk M, Coulter RWS, Egan JE, Fisk S, Reuel Friedman M, Tula M, Kinsky S. Harm reduction principles for healthcare settings. Harm Reduct J. 2017;14(1):70. 10.1186/s12954-017-0196-4. PMC5655864 29065896

[29] Hervera B, Seo G, Bartholomew TS, Chueng TA, Suarez E, Forrest DW, Hernandez S, Rodriguez AE, Tookes HE, Doblecki-Lewis S, Serota DP. Implementation of an integrated infectious disease and substance use disorder team for injection drug use-associated infections: A qualitative study. Addict Sci Clin Pract. 2023;18(1):8. 10.1186/s13722-023-00363-4. PMC9902242 36747268

[30] Hillyard M, Cockbain B, Drummond C, Rimes K, Roberts E. Barriers and facilitators to accessing addiction treatment services for sexual minority (LGBQ+) people: Systematic review. Br J Psychiatry: J Mental Sci. 2026;1–11. 10.1192/bjp.2026.10589. PMC7619179 41906218

[31] Holmes SM. Fresh Fruit, Broken Bodies: Migrant Farmworkers in the United States. 1st ed. University of California Press; 2013. https://www.jstor.org/stable/. 10.1525/j.ctt7zw45x.

[32] Hser Y-I, Longshore D, Anglin MD. The life course perspective on drug use: A conceptual framework for understanding drug use trajectories. Eval Rev. 2007;31(6):515–47. 10.1177/0193841X07307316. 17986706

[33] Hughto JMW, Quinn EK, Dunbar MS, Rose AJ, Shireman TI, Jasuja GK. Prevalence and Co-occurrence of Alcohol, Nicotine, and Other Substance Use Disorder Diagnoses Among US Transgender and Cisgender Adults. JAMA Netw Open. 2021;4(2):e2036512. 10.1001/jamanetworkopen.2020.36512. PMC7862992 33538824

[34] Hughto JMW, Restar AJ, Wolfe HL, Gordon LK, Reisner SL, Biello KB, Cahill SR, Mimiaga MJ. Opioid pain medication misuse, concomitant substance misuse, and the unmet behavioral health treatment needs of transgender and gender diverse adults. Drug Alcohol Depend. 2021;222:108674. 10.1016/j.drugalcdep.2021.108674. PMC8058310 33773869

[35] Hussen SA, Camp DM, Jones MD, Patel SA, Crawford ND, Holland DP, Cooper HLF. Exploring influences on methamphetamine use among Black gay, bisexual and other men who have sex with men in Atlanta: A focus group study. Int J Drug Policy. 2021;90:103094. 10.1016/j.drugpo.2020.103094. PMC8380493 33429161

[36] Javanbakht M, Rosen AD, Moore E, Shoptaw S, Gorbach PM. Brief Report: Intersecting Vulnerabilities: The Role of Housing Instability and Methamphetamine Use on HIV Acquisition. J Acquir Immune Defic Syndr. 2026;101(6):575–9. 10.1097/QAI.0000000000003836. 41587021

[37] Jenkins WD, Beach LB, Schneider J, Friedman SR, Pho MT, Walters S, Ezell J, Young AM, Hennessy C, Miller WC, Go VF, Sun C, Seal DW, Westergaard RP, Crane HM, Fredericksen RJ, Ruderman SA, Fletcher S, Ma J, Winer M. Sexual identity, sexual behavior, and drug use behaviors among people who use drugs in the rural U.S. J Subst Use Addict Treat. 2025;171:209629. 10.1016/j.josat.2025.209629. PMC13255774 39875013

[38] Jerome RC, Halkitis PN. An Exploratory Investigation of Treatment Strategies for Black, Gay, Bisexual, and Heterosexual Men-Who-Have-Sex- With-Men Who Use Methamphetamine. J LGBT Issues Couns. 2014;8(1):2–24. 10.1080/15538605.2014.853636.

[39] Kampman K, Jarvis M. American Society of Addiction Medicine (ASAM) National Practice Guideline for the Use of Medications in the Treatment of Addiction Involving Opioid Use. J Addict Med. 2015;9(5):358–67. 10.1097/ADM.0000000000000166. PMC4605275 26406300

[40] Knight R, Karamouzian M, Carson A, Edward J, Carrieri P, Shoveller J, Fairbairn N, Wood E, Fast D. Interventions to address substance use and sexual risk among gay, bisexual and other men who have sex with men who use methamphetamine: A systematic review. Drug Alcohol Depend. 2019;194:410–29. 10.1016/j.drugalcdep.2018.09.023. 30502543

[41] Komaromy M, Stone A, Peterson A, Gott J, Koenig R, Taylor JL. Facilitating exit from encampments: Combining low-barrier transitional housing with stabilizing treatment for substance related problems. Addict Sci Clin Pract. 2023;18(1):66. 10.1186/s13722-023-00420-y. PMC10601141 37884986

[42] Krasnova A, Diaz JE, Philbin MM, Mauro PM. Disparities in substance use disorder treatment use and perceived need by sexual identity and gender among adults in the United States. Drug Alcohol Depend. 2021;226:108828. 10.1016/j.drugalcdep.2021.108828. PMC8477369 34325282

[43] Larochelle MR, Bernson D, Land T, Stopka TJ, Wang N, Xuan Z, Bagley SM, Liebschutz JM, Walley AY. Medication for Opioid Use Disorder After Nonfatal Opioid Overdose and Association With Mortality: A Cohort Study. Ann Intern Med. 2018;169(3):137–45. 10.7326/M17-3107. PMC6387681 29913516

[44] McGeough BL, Greenwood E (M), Cohen NL, Wootton AR. (2023). Integrating SMART Recovery and Mental Health Services to Meet the Needs and Goals of LGBTQ Individuals Experiencing Substance Use–Related Problems. Families in Society: The Journal of Contemporary Social Services, 104(2), 222–233. 10.1177/10443894221124621

[45] Mendola A, Gibson RL. Addiction, 12-Step Programs, and Evidentiary Standards for Ethically and Clinically Sound Treatment Recommendations: What Should Clinicians Do? AMA J Ethics. 2016;18(6):646–55. 10.1001/journalofethics.2016.18.6.sect1-1606. 27322999

[46] Moore KA, Young MS, Barrett B, Ochshorn E. A 12-Month Follow-Up Evaluation of Integrated Treatment for Homeless Individuals With Co-Occurring Disorders. J Social Service Res. 2009;35(4):322–35. 10.1080/01488370903110829.

[47] National Academies of Sciences, Division E, H. and, Policy M, Disorder B. C. on M.-A. T. for O. U., Mancher M., & Leshner A. I. (2019). Barriers to Broader Use of Medications to Treat Opioid Use Disorder. In Medications for Opioid Use Disorder Save Lives. National Academies Press (US). https://www.ncbi.nlm.nih.gov/books/NBK541389/30896911

[48] Padgett DK, Stanhope V, Henwood BF, Stefancic A. Substance Use Outcomes Among Homeless Clients with Serious Mental Illness: Comparing Housing First with Treatment First Programs. Commun Ment Health J. 2011;47(2):227–32. 10.1007/s10597-009-9283-7. PMC2916946 20063061

[49] Paschen-Wolff MM, DeSousa A, Paine EA, Hughes TL, Campbell ANC. Experiences of and recommendations for LGBTQ+-affirming substance use services: An exploratory qualitative descriptive study with LGBTQ+ people who use opioids and other drugs. Subst Abuse Treat Prev Policy. 2024;19:2. 10.1186/s13011-023-00581-8. PMC10765665 38172902

[50] Paschen-Wolff MM, Velasquez R, Aydinoglo N, Campbell ANC. Simulating the experience of searching for LGBTQ-specific opioid use disorder treatment in the United States. J Subst Abuse Treat. 2022;140:108828. 10.1016/j.jsat.2022.108828. 35749919

[51] Philbin MM, Greene ER, Martins SS, LaBossier N, Mauro PM. Medical, Nonmedical, and Illegal Stimulant Use by Sexual Identity and Gender. Am J Prev Med. 2020;59(5):686–96. 10.1016/j.amepre.2020.05.025. PMC7577928 32981768

[52] Quesada J, Hart LK, Bourgois P. Structural vulnerability and health: Latino migrant laborers in the United States. Med Anthropol. 2011;30(4):339–62. 10.1080/01459740.2011.576725. PMC3146033 21777121

[53] Reback CJ, Shoptaw S. Development of an evidence-based, gay-specific cognitive behavioral therapy intervention for methamphetamine-abusing gay and bisexual men. Addict Behav. 2014;39(8):1286–91. 10.1016/j.addbeh.2011.11.029. PMC3326187 22169619

[54] Rhodes T, Wagner K, Strathdee SA, Shannon K, Davidson P, Bourgois P. (2012). Structural Violence and Structural Vulnerability Within the Risk Environment: Theoretical and Methodological Perspectives for a Social Epidemiology of HIV Risk Among Injection Drug Users and Sex Workers. In O’Campo P. & Dunn J. R., editors, Rethinking Social Epidemiology: Towards a Science of Change (pp. 205–230). Springer Netherlands. 10.1007/978-94-007-2138-8_10

[55] Rosenwohl-Mack S, Heeney M, Samuel L, Anderson E, Herring AA, Sirey LR, Peterson D, Bazazi AR, Snyder H, Suen LW. Outpatient initiation of direct-to-inject buprenorphine. Drug Alcohol Depend. 2026;278:113011. 10.1016/j.drugalcdep.2025.113011. PMC13271074 41442975

[56] Santos G-M, Coffin P, Santos D, Huffaker S, Matheson T, Euren J, DeMartini A, Rowe C, Hahn JA, Vlahov D, Vittinghoff E, Batki SL. Feasibility, Acceptability, and Tolerability of Targeted Naltrexone for Nondependent Methamphetamine-Using and Binge-Drinking Men Who Have Sex with Men. JAIDS J Acquir Immune Defic Syndr. 2016;72(1):21. 10.1097/QAI.0000000000000922. PMC4837026 26674372

[57] Schepis TS, West BT, Ford JA, Veliz PT, McCabe SE. Differences in Substance Use and Mental Health by Sexual Identity Among U.S. Adolescents and Young Adults. LGBT Health. 2026;13(3):157–66. 10.1177/23258292261419535. PMC13535610 41665462

[58] Schuler MS, Prince DM, Collins RL. Disparities in Social and Economic Determinants of Health by Sexual Identity, Gender, and Age: Results from the 2015–2018 National Survey on Drug Use and Health. LGBT Health. 2021;8(5):330–9. 10.1089/lgbt.2020.0390. PMC8252906 34101498

[59] Schuler MS, Rice CE, Evans-Polce RJ, Collins RL. Disparities in substance use behaviors and disorders among adult sexual minorities by age, gender, and sexual identity. Drug Alcohol Depend. 2018;189:139–46. 10.1016/j.drugalcdep.2018.05.008. PMC6083846 29944989

[60] Serota DP, Rosenbloom L, Hervera B, Seo G, Feaster DJ, Metsch LR, Suarez E, Chueng TA, Hernandez S, Rodriguez AE, Tookes HE, Doblecki-Lewis S, Bartholomew TS. Integrated Infectious Disease and Substance Use Disorder Care for the Treatment of Injection Drug Use-Associated Infections: A Prospective Cohort Study With Historical Control. Open Forum Infect Dis. 2023;10(1):ofac688. 10.1093/ofid/ofac688. PMC9830545 36632415

[61] Shoptaw S, Reback CJ, Larkins S, Wang P-C, Rotheram-Fuller E, Dang J, Yang X. Outcomes using two tailored behavioral treatments for substance abuse in urban gay and bisexual men. J Subst Abuse Treat. 2008;35(3):285–93. 10.1016/j.jsat.2007.11.004. 18329226

[62] Shoptaw S, Reback CJ, Peck JA, Yang X, Rotheram-Fuller E, Larkins S, Veniegas RC, Freese TE, Hucks-Ortiz C. Behavioral treatment approaches for methamphetamine dependence and HIV-related sexual risk behaviors among urban gay and bisexual men. Drug Alcohol Depend. 2005;78(2):125–34. 10.1016/j.drugalcdep.2004.10.004. 15845315

[63] Sinclair DL, Chantry M, De Ruysscher C, Magerman J, Nicaise P, Vanderplasschen W. Recovery-supportive interventions for people with substance use disorders: A scoping review. Front Psychiatry. 2024;15:1352818. 10.3389/fpsyt.2024.1352818. PMC10991812 38577404

[64] Srivastava A, Rhoades H, Prost SG, Hall WJ. Houselessness, Sexual Orientation, and Mental Health Among Adults in United States Prisons. Sexuality Res Social Policy. 2024;21(3):960–8. 10.1007/s13178-023-00868-2.

[65] Steiner G, Baral S, Riley ED, Shoptaw S, Chamie G, Roberts K, Suchman L, Knight K, Gandhi M, Coffin P, Appa A. Harm Reduction Contingency Management for Stimulant Use Reduction and Antiretroviral Therapy Adherence in HIV Primary Care: Protocol for an Implementation Effectiveness Study. JMIR Res Protocols. 2025;14:e67292. 10.2196/67292. PMC12402737 40825537

[66] Substance Abuse and Mental Health Services Administration. (2024). 2023 National Survey on Drug Use and Health: Among the Lesbian, Gay, Bisexual, or Some Other Term (LGB+) Population Aged 12 or Older. https://www.samhsa.gov/data/sites/default/files/reports/rpt53159/2023-nsduh-pop-slides-lbgplus.pdf

[67] Substance Abuse and Mental Health Services Administration. (2025). Key Substance Use and Mental Health Indicators in the United States: Results from the 2024 National Survey on Drug Use and Health. https://www.samhsa.gov/data/data-we-collect/nsduh-national-survey-drug-use-and-health/national-releases

[68] Trivedi MH, Walker R, Ling W, Cruz Adela, Sharma G, Carmody T, Ghitza UE, Wahle A, Kim M, Shores-Wilson K, Sparenborg S, Coffin P, Schmitz J, Wiest K, Bart G, Sonne SC, Wakhlu S, Rush AJ, Nunes EV, Shoptaw S. Bupropion and Naltrexone in Methamphetamine Use Disorder. N Engl J Med. 2021;384(2):140–53. 10.1056/NEJMoa2020214. PMC8111570 33497547

[69] Tsemberis S, Gulcur L, Nakae M. Housing First, consumer choice, and harm reduction for homeless individuals with a dual diagnosis. Am J Public Health. 2004;94(4):651–6. 10.2105/ajph.94.4.651. PMC1448313 15054020

[70] Vederhus J-K, Høie M, Birkeland B. One size doesn’t fit all: A thematic analysis of interviews with people who have stopped participating in Narcotics Anonymous in Norway. Addict Sci Clin Pract. 2020;15(1):18. 10.1186/s13722-020-00191-w. PMC7247232 32448354

[71] Wakeman SE, Larochelle MR, Ameli O, Chaisson CE, McPheeters JT, Crown WH, Azocar F, Sanghavi DM. Comparative Effectiveness of Different Treatment Pathways for Opioid Use Disorder. JAMA Netw Open. 2020;3(2):e1920622. 10.1001/jamanetworkopen.2019.20622. PMC11143463 32022884

[72] Wenger LD, Morris T, Knight KR, Megerian CE, Davidson PJ, Suen LW, Majano V, Lambdin BH, Kral AH. Radical hospitality: Innovative programming to build community and meet the needs of people who use drugs at a government-sanctioned overdose prevention site in San Francisco, California. Int J Drug Policy. 2024;126:104366. 10.1016/j.drugpo.2024.104366. PMC11160962 38492432

[73] Zhang C, Tang Y, Kniffen W, Liu Y. Exploring Social and Structural Determinants of Substance Use Among Sexual and Gender Minority Adults in the United States. J Int Association Providers AIDS Care. 2025;24:23259582251409855. 10.1177/23259582251409855. PMC12741260 41432589

